# Relationally specific sexual identity concealment and loneliness among sexual and gender minority women in Japan: A culturally situated analysis

**DOI:** 10.1002/pcn5.70280

**Published:** 2026-01-08

**Authors:** Maya Hayakawa, Junichi Fujita, Yusuke Saigusa, Yuriko Tanabe, Akitoyo Hishimoto, Takeshi Asami

**Affiliations:** ^1^ Department of Psychiatry Yokohama City University Graduate School of Medicine Yokohama Japan; ^2^ Department of Child Psychiatry Yokohama City University Hospital Yokohama Japan; ^3^ Department of Biostatistics Yokohama City University School of Medicine Yokohama Japan; ^4^ Department of Nursing Yokohama City University School of Medicine Yokohama Japan; ^5^ Department of Psychiatry Kobe University Graduate School of Medicine Kobe City Japan

**Keywords:** concealment, cultural norms, loneliness, relational context, sexual and gender minority women

## Abstract

**Aim:**

To examine whether sexual identity concealment is differentially associated with loneliness across relational domains—parents and heterosexual friends—among sexual and gender minority (SGM) women in Japan, and to interpret these patterns within cultural norms.

**Methods:**

We conducted a cross‐sectional, self‐administered paper questionnaire study of SGM women in Japan (*N* = 166). Loneliness was assessed using the Japanese version of the Revised UCLA Loneliness Scale. Concealment was measured with binary items (1 = concealment, 0 = disclosure) for two domains. Multiple linear regressions estimated domain‐specific associations with loneliness, adjusting for age; sexual orientation (reference = lesbian); gender identity (cisgender vs. non‐cisgender); marital status (partnered vs. single); psychiatric service use in the past year (yes/no); lifetime substance use (ever vs. never); lesbian, gay, bisexual, transgender, and queer (LGBTQ)–based victimization (yes/no); and perceived prejudice (yes/no).

**Results:**

Concealment from heterosexual friends was associated with higher loneliness, whereas concealment from parents showed no clear association with loneliness. Bisexual identity, compared with lesbian identity, and past‐year psychiatric service use were also related to greater loneliness.

**Conclusion:**

Sexual identity concealment is not monolithic; its association with loneliness depends on relational context. Peer‐based concealment appears more consequential for loneliness than parent‐directed concealment among SGM women in Japan. Interventions should address culturally situated norms in peer relationships, promote alternative pathways to connection, and avoid pathologizing strategic silence while mitigating its emotional costs.

## INTRODUCTION

In cultures that prioritize relational harmony, social expectations often constrain personal expression expectations.^1^ Such dynamics have been described in various non‐Western contexts, including other Asian and Muslim‐majority societies, where concerns regarding relational obligations and family reputation are salient. Japan is one such instance, where individuals are socialized to avoid imposing on others or disturbing collective equilibrium and where concerns regarding family reputation and social appearance carry a significant weight.

These relational pressures are especially salient for women. Qualitative studies describe deliberate silence and selective communication, particularly within mother–daughter relationships, to maintain domestic harmony.^2^ While such practices are culturally adaptive means of keeping the family peace, they can also limit emotional openness within key relationships.^3^

In many Asian and Muslim‐majority contexts, sexual and gender minority (SGM) individuals similarly navigate tensions between family obligations, cultural or religious expectations, and disclosure risk.^4^, ^5^ In such environments, openness about same‐sex attraction can be potentially disruptive to family harmony and social standing, even in the absence of explicit condemnation.

Concealment, defined as the intentional withholding of personal identity, consequently becomes a relational strategy for sustaining harmony, but at the cost of reduced emotional reciprocity. Although identity concealment has been linked to psychological distress and loneliness,^6^, ^7^ most studies have treated it as an individual coping response to stigma. The variations in emotional correlates of concealment across relational domains remain understudied.

In Japan, family relationships emphasize harmony and obligation, while friendships are grounded in reciprocity and mutual openness.^8^ Thus, the emotional implications of concealment may vary in these distinct relational contexts.

The present study explores the differential associations of loneliness with concealment from parents and from heterosexual friends among SGM women in Japan. The analytical focus on relational domains rather than individual traits aims to clarify the interpersonal context that is more closely linked to loneliness. The exploratory approach offers culturally embedded insights into the functions of concealment, both as a relational adaptation and a potential source of emotional cost.

## METHODS

### Study design and setting

This cross‐sectional study employed venue‐based community recruitment conducted in Tokyo, Japan, between May 2015 and March 2016. Participants were approached at the Tokyo Rainbow Pride 2015 and at a lesbian community space.

### Participants

**Figure 1:**
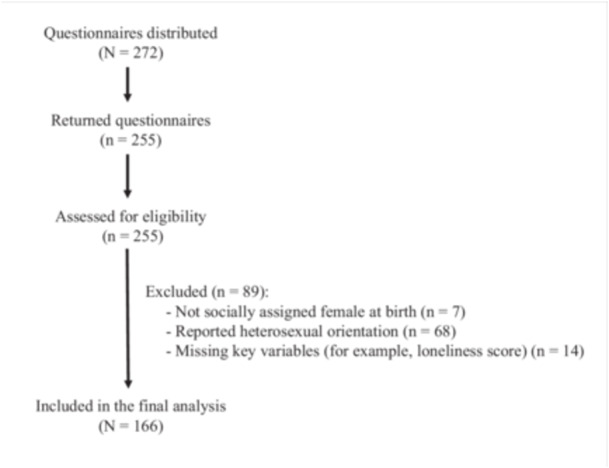
Study flow diagram showing participant recruitment, exclusion, and final analytic sample (*N* = 166). Questionnaires distributed (*N* = 272); returned (*n* = 255); excluded (*n* = 89: not sex assigned female at birth, *n* = 7; heterosexual identity, *n* = 68; missing outcome, *n* = 14); included (*n* = 166). Participants were recruited from Tokyo Rainbow Pride and a lesbian community space between May 2015 and March 2016.

We recruited participants using an anonymous, self‐administered paper questionnaire targeting SGM women in Japan. Eligibility criteria included being assigned female at birth, aged 18 years or older, and currently residing in Japan. Measures and analytic procedures are described below. The participant flow is shown in Figure 1.

### Ethical considerations

The study was approved by the Human Genome and Genetic Research Ethics Committee of Yokohama City University (Approval Nos. A151126016 and A190100005). Written and oral information on the study was provided, and informed consent was obtained from the participants before they completed the questionnaire. All procedures adhered to the 2013 Declaration of Helsinki.

### Measures

#### Demographics

Demographic data collected included sexual orientation, gender identity, age, marital status, lifetime substance use, past‐year psychiatric service use, lesbian, gay, bisexual, transgender, and queer (LGBTQ)–based victimization, and perceived prejudice. Sexual orientation was assessed using a self‐identification item (response options: lesbian [reference], bisexual, queer, asexual, or other). Gender identity was assessed by asking whether participants identified as a cisgender or non‐cisgender woman (with an “other” free‐text option). Eligibility required being socially assigned female at birth. Age was recorded in years and analyzed as a continuous variable; for descriptive reporting, age groups were 18–19, 20–29, 30–39, and ≥40 years. Marital status was coded as partnered (reference) versus single. Lifetime substance use was coded as ever versus never. Psychiatric service use was coded for the past year (yes/no). LGBTQ‐based victimization and perceived prejudice were each coded yes/no.

#### Loneliness

The primary outcome was loneliness, analyzed as a continuous total score on the Japanese version of the Revised UCLA Loneliness Scale (R‐UCLALS‐J).^9^, ^10^ This 20‐item scale assesses subjective feelings of social isolation on a 4‐point Likert scale (1 = Never to 4 = Often), with 10 items reverse‐scored. Item scores were summed to yield a total score ranging from 20 to 80, with higher scores indicating greater loneliness. Prior studies have confirmed the excellent internal consistency (*α* > 0.90–0.94) of the R‐UCLALS‐J. The Cronbach's *α* in the present sample was 0.87. Example items for loneliness include “I lack companionship,” “No one really knows me well,” and a positively worded item such as “I am an outgoing person” (reverse‐scored).

#### Concealment

Sexual identity concealment was evaluated in two relational domains: with parents and with heterosexual friends. The items (originally written in Japanese) asked the following questions: “Have you ever disclosed your sexual identity (e.g., that you are lesbian, bisexual, queer, etc.) to your parents?” and “Have you ever disclosed your sexual identity (e.g., that you are lesbian, bisexual, queer, etc.) to your heterosexual friends?” (response options: yes/no).

For the analyses, each item was recoded to reflect sexual identity concealment in the corresponding domain: responses indicating that the participant had never disclosed her sexual identity were coded as 1 (concealment present); otherwise, they were coded as 0 (no concealment). Therefore, each domain was treated as a single binary indicator of whether the participant concealed her sexual identity from “her parents” and “heterosexual friends in general.” The “parents” item referred to parents collectively and did not differentiate between mothers and fathers or other caregiver constellations (e.g., single parents, step‐parents). The item for heterosexual friends did not differentiate between close and distant friends or between more‐ and less‐trusted confidants, and neither item enabled partial or selective disclosure (e.g., disclosing to one parent but not the other, or to some but not all friends). The indicator for friends served as the primary explanatory variable, whereas that for parents was included for comparison within the same model.

#### LGBTQ‐based victimization and perceived prejudice

Two binary items assessed distal minority stressors. LGBTQ‐based victimization was measured with a yes/no question asking participants' experiences of direct harassment, discrimination, or violence attributed to their sexual orientation or gender identity. Perceived prejudice was measured with a yes/no question asking participants' direct or indirect experience of negative remarks or discriminatory attitudes toward LGBTQ people, even if not personally targeted.

#### Covariates

We adjusted for age (years), sexual orientation (reference = lesbian), gender identity (cisgender vs. non‐cisgender), marital status (partnered vs. single), psychiatric service use in the past year (yes/no), lifetime substance use (ever vs. never), LGBTQ‐based victimization (yes/no), and perceived prejudice (yes/no). All categorical covariates were dummy‐coded with the reference categories indicated. Sexual orientation distinguished lesbian (reference) from bisexual and other sexual minority identities; gender identity distinguished cisgender women (reference) from non‐cisgender women. Covariates were selected a priori based on prior literature linking demographic factors and minority stress exposures to both concealment and loneliness. Target‐specific predictors (concealment from parents and from heterosexual friends) were entered simultaneously in all models.

### Data analysis

All statistical analyses were performed using spss (version 30.0; IBM Corp., Armonk, NY, USA). Only cases with complete data were analyzed; participants with missing data on any study variable were excluded (listwise deletion), and no imputations were performed. Descriptive statistics (mean, standard deviation, and frequency) were calculated to summarize sample characteristics and concealment patterns, with separate calculations for concealment from parents and from heterosexual friends. To examine unadjusted differences in loneliness, independent‐sample *t*‐tests compared loneliness scores between those who concealed and disclosed within each relational domain. Welch's correction was applied when variances were unequal, and values were reported with effect sizes (Cohen's *d*) and 95% confidence intervals (CIs). Bivariate correlations between age and loneliness were calculated using Pearson's *r*. Finally, multiple linear regression analyses were performed to estimate the adjusted associations between relational concealment and loneliness. Both concealment indicators (parents and heterosexual friends) were entered simultaneously, controlling for age (years), sexual orientation (reference = lesbian), gender identity (cisgender vs. non‐cisgender), marital status (partnered vs. single), psychiatric service use in the past year (yes/no), lifetime substance use (ever vs. never), LGBTQ‐based victimization (yes/no), and perceived prejudice (yes/no). Standardized coefficients (*β*), 95% CIs, *R* ^2^, adjusted *R* ^2^, and *F* statistics were reported. Model assumptions of linearity, homoscedasticity, and normality of residuals were evaluated. Multicollinearity was examined using variance inflation factors. All analyses were two‐tailed with a significance level of *α* = 0.05.

## RESULTS

Questionnaires were distributed to 272 individuals, of whom 255 returned the survey (93.8%). We excluded 7 participants who were not assigned female at birth, 68 who identified as heterosexual, and 14 with missing outcome data on the R‐UCLALS‐J. The final analytic sample comprised 166 participants who provided complete data on all study variables (Figure 1).

Participants' age was 18–56 years (mean 28.8 years, SD = 8.7; median = 26; interquartile range = 22–34; mode = 21). Regarding sexual orientation, 39.2% identified as lesbian, 44.6% as bisexual, 7.8% as queer, and 2.4% as asexual or other identities. In terms of gender identity, 6.0% identified as non‐cisgender women. Descriptive statistics and bivariate comparisons are presented in Table S1, and regression analysis results are summarized in Table 1.

**Table 1:** Multiple linear regression predicting loneliness from concealment.

	*B*	SE	*β*	*p*	95% CI for *B*
Intercept	38.03	4.02	–	<0.001	30.09, 45.98
Concealment from heterosexual friends	6.03	2.07	0.22	0.004	1.95, 10.11
Concealment from parents	−1.72	1.74	−0.08	0.326	−5.16, 1.73
LGBTQ‐based victimization (yes)	1.62	1.73	0.07	0.352	−1.80, 5.03
Perceived prejudice (yes)	1.69	2.89	0.04	0.559	−4.01, 7.40
Psychiatric service use (past year, yes)	6.68	1.79	0.28	<0.001	3.15, 10.21
Lifetime substance use (yes)	2.20	2.94	0.06	0.455	−3.60, 8.01
Partnership (partnered vs. single)	−3.08	1.57	−0.14	0.051	−6.18, 0.02
Age (years)	−0.10	0.09	−0.08	0.296	−0.28, 0.09
Bisexual (vs. lesbian)	3.36	1.65	0.15	0.043	0.11, 6.61
Gender identity (non‐cisgender vs. cisgender)	2.44	3.48	0.05	0.484	−4.43, 9.32

*Note*: Model summary: *R* ^2^ = 0.23, adjusted *R* ^2^ = 0.18. The model adjusted for age (years), sexual orientation (reference = lesbian), gender identity (cisgender vs. non‐cisgender), marital status (partnered vs. single), psychiatric service use in the past year (yes/no), lifetime substance use (ever vs. never), LGBTQ‐based victimization (yes/no), and perceived prejudice (yes/no).

Abbreviations: *β*, standardized coefficient; *B*, unstandardized coefficient; CI, confidence interval; LGBTQ, lesbian, gay, bisexual, transgender, and queer; SE, standard error.

**p < 0.01.

In the fully adjusted model (Table 1), concealment from heterosexual friends was associated with greater loneliness compared with disclosure (*β* = 0.22, *p* = 0.004), whereas concealment from parents was not associated with loneliness (*β* = −0.08, *p* = 0.33). Past‐year psychiatric service use (*β* = 0.28, *p* < 0.001) and bisexual identity compared with lesbian identity (*β* = 0.15, *p* = 0.043) were also associated with higher loneliness; other covariates were not significant. The model accounted for approximately 23% of the variance in loneliness (*R* ^2^ = 0.23, adjusted *R* ^2^ = 0.18). Sensitivity analyses excluding the small non‐cisgender subgroup (*n* = 10) yielded highly similar results: the association between concealment from heterosexual friends and loneliness remained substantial (*B* = 5.12, SE = 2.12, *β* = 0.19, *p* = 0.017), whereas parental concealment remained non‐significant (*B* = −1.80, SE = 1.73, *β* = −0.08, *p* = 0.30).

Questionnaires were distributed to 272 individuals, of whom 255 returned the survey (93.8%). We excluded 7 participants who were not assigned female at birth, 68 who identified as heterosexual, and 14 with missing outcome data on the R‐UCLALS‐J. The final analytic sample comprised 166 participants who provided complete data on all study variables (Figure 1).

Participants' age was 18–56 years (mean 28.8 years, SD = 8.7; median = 26; interquartile range = 22–34; mode = 21). Regarding sexual orientation, 39.2% identified as lesbian, 44.6% as bisexual, 7.8% as queer, and 2.4% as asexual or other identities. In terms of gender identity, 6.0% identified as non‐cisgender women. Descriptive statistics and bivariate comparisons are presented in Table S1, and regression analysis results are summarized in Table 1.

In the fully adjusted model (Table 1), concealment from heterosexual friends was associated with greater loneliness compared with disclosure (*β* = 0.22, *t*(155) = 2.92, *p* = 0.004), whereas concealment from parents was not associated with loneliness (*β* = −0.08, *t*(155) = − 0.99, *p* = 0.33). Past‐year psychiatric service use (*β* = 0.28, *t*(155) = 3.74, *p* < 0.001) and bisexual identity compared with lesbian identity (*β* = 0.15, *t*(155) = 2.04, *p* = 0.043) were also associated with higher loneliness; other covariates were not significant. The model explained approximately 23% of the variance in loneliness (*R* ^2^ ≈ 0.23, adjusted *R* ^2^ ≈ 0.18) and showed significant overall fit, *F*(10, 155) ≈ 4.5, *p* < 0.001. Sensitivity analyses excluding the small non‐cisgender subgroup (*n* = 10) yielded highly similar results: the association between concealment from heterosexual friends and loneliness remained substantial (*B* = 5.12, SE = 2.12, *β* = 0.19, *p* = 0.017), whereas parental concealment remained non‐significant (*B* = −1.80, SE = 1.73, *β* = −0.08, *p* = 0.30).

## DISCUSSION

This study empirically examined the differential associations between relational sexual identity concealment and loneliness among SGM women in Japan, with concealment from parents and heterosexual friends as the distinct domains. The analysis revealed that sexual identity concealment within friendships, but not within family relationships, was significantly associated with greater loneliness. These patterns remained essentially unchanged in the sensitivity analyses excluding the small non‐cisgender subgroup (*n* = 10), in which concealment from heterosexual friends remained positively associated with loneliness, whereas parental concealment remained unrelated to loneliness. Although modest in magnitude, the adjusted model explained 23% of the variance in loneliness (adjusted *R* ^2^ = 0.18), suggesting that concealment operates alongside other determinants.

This relational asymmetry suggests that sexual identity concealment is not a uniform stressor but rather a contextually negotiated practice among SGM women in Japan. Contrary to the results of minority stress frameworks conceptualizing nondisclosure of sexual identity as inherently maladaptive,^6^, ^7^ the lack of an association between parental sexual identity concealment and loneliness indicates that silence within families does not necessarily signify emotional withdrawal. In some cases, nondisclosure may function as a culturally intelligible form of care, given that restraint and selective talk are embedded within Japanese interdependent family systems, where harmony often supersedes individual expression.^11^ However, considering that the average age of our participants was approximately 30 years and many were likely to be financially and emotionally independent from their parents, concealment from parents may also reflect other relational patterns. Avoiding conversations regarding sexual identity can indicate that relationships are relatively distant or not central to everyday life, that individuals prefer to keep parents uninvolved in this domain, or that parents are not considered as confidants or sources of advice regarding sexuality, as suggested by research on topic avoidance and (non)disclosure in close relationships.^12^, ^13^ As our dichotomous measure of disclosure did not evaluate relationship quality or reasons for nondisclosure, we cannot empirically distinguish among these possibilities. Nonetheless, the present findings extend the communication privacy management theory^14^ by illustrating that the same behavioral choice—remaining silent with parents—can carry distinct meanings depending on cultural norms and the degree of dependency and closeness within families.

This perspective highlights the paradox of sexual identity concealment: It can be protective in some relationships but isolating in others. In friendships, which are founded on reciprocity and mutual openness,^15^ silence about sexual identity can be interpreted as distance or mistrust. This creates relational dissonance where individuals concealing their sexual identity from close friends undermine the sense of intimacy that defines friendship instead of preserving it. This dynamic resonates with the concept of loneliness as a qualitative mismatch between desired and actual social connection.^16^ By situating this finding within interdependence cultural models, concealment in friendships violates the implicit expectation of shared emotional transparency that sustains peer intimacy in collectivist contexts. Sensitivity analyses (multicollinearity checks, influence diagnostics, and alternative coding) produced substantively unchanged results: the association for friends remained positive, and the association for parent remained null.

Gendered cultural expectations further complicate the scenario. In Japan, femininity has long been associated with empathy, emotional labor, and the responsibility of maintaining social harmony.^17^, ^18^, ^19^, ^20^ When women who internalize these ideals engage in sexual identity concealment, the act conflicts with their self‐concept as emotionally attuned and transparent, creating incongruence that evokes guilt or self‐blame, intensifying loneliness despite their success in maintaining relational order. Therefore, concealment functions not only as a boundary‐regulating practice but also as a gendered emotional task shaped by cultural expectations of care and relational maintenance. This perspective bridges gendered emotion norms with minority stress processes, signifying that emotional dissonance may emerge less from concealment itself than from a perceived failure to meet gendered expectations of emotional reciprocity.

These findings refine existing theoretical frameworks by showing that the emotional costs of sexual identity concealment are shaped by relational norms rather than by nondisclosure itself. By integrating minority stress and communication privacy management theories within a cultural framework, concealment can be reconceptualized not as a static coping mechanism but as a relationally embedded negotiation process. Western models of minority stress often privilege openness about sexual identity as a universal good and may implicitly construe silence as pathology. However, in cultures that value social harmony and indirectness, selective silence about sexual identity is a morally intelligible form of connection rather than a disconnection. This distinction may be crucial for developing culturally responsive models of psychological well‐being.

In summary, sexual identity concealment cannot be understood apart from the relational scripts and emotional economies in which it is enacted. In the Japanese context, silence about sexual identity may simultaneously protect social bonds and constrain authentic expression, creating a tension that is both culturally adaptive and psychologically costly. This duality reveals concealment not as an absence of openness, but as an active relational negotiation that balances safety, belonging, and authenticity.

Recent sexual minority mental health and cross‐cultural and comparative psychology studies further support a relational view of sexual identity concealment, indicating that the meaning of self‐concealment of sexual minority status is shaped by cultural relational values.^21^, ^22^ In interdependent or high‐context societies, emotional restraint and selective privacy can promote belonging and moral appropriateness, not isolation. Such findings suggest that concealment can be a relationally coherent strategy within local systems of social attunement and moral responsibility.^23^, ^24^

These insights emphasize the importance of culturally responsive approaches to sexual identity disclosure in clinical practice. Recent psychotherapy research conceptualizes silence as co‐constructed in interaction rather than solely as an individual defense^25^, ^26^ and cultural clinical psychology emphasizes that privacy boundaries are co‐constructed through relational negotiation and cannot be understood solely as individual defense mechanisms. Accordingly, clinicians and medical providers working with SGM patients should recognize both the ethical dimensions of silence about sexual identity and the agency embedded in privacy regulation.^27^, ^28^ In practice, this may involve collaboratively identifying with each patient the relationships in which she feels most comfortable being open about her sexual identity such as with specific family members, heterosexual friends, or members of LGBTQ communities—rather than assuming that disclosure to parents is invariably the primary or ultimate objective. Such conversations can support small, contextually appropriate steps toward greater authenticity where desired while respecting the patient's wish to maintain privacy in other relationships. Rather than pathologizing silence about sexual identity as avoidance, clinicians may find it useful to view it as a relational act that can help maintain dignity and situational safety within specific cultural contexts.

At a broader level, these findings raise questions about the conceptualization of openness and authenticity in global mental health models. While Western frameworks often equate psychological health with transparency about one's inner self and identities, this assumption may overlook relational cultures where modesty and discretion sustain social cohesion.^21^, ^29^ A culturally integrative perspective would instead regard sexual identity concealment and disclosure as adaptive strategies along a continuum of relational maintenance.

Future studies should examine the impact of shifting norms of gender and intimacy on the emotional meaning of silence about sexual identity in Japan and other societies undergoing social change. By recognizing the relational ethics of sexual identity privacy, future psychological investigations can move beyond disclosure‐centric frameworks and toward a more context‐sensitive understanding of relational connection and well‐being.

Collectively, these findings should be interpreted in view of several limitations. The major limitation of this study is that sexual identity concealment in each relational domain was evaluated using single binary yes/no items. For parents, the item asked whether participants had ever disclosed their sexual identity to “their parents,” treating them as a collective category and not distinguishing between mothers and fathers or more complex family structures (e.g., single parents, step‐parents). For heterosexual friends, the item asked whether participants concealed their sexual identity from “heterosexual friends in general.” This approach precluded the differentiation of close, trusted friends from more distant or less‐reliable acquaintances and could not capture partial or selective disclosure (e.g., disclosing to one parent but not the other, or to some but not all friends). Consequently, our measure likely conflates heterogeneous relational contexts and underestimates the nuance of relationally specific concealment. Therefore, the observed association between concealment from heterosexual friends and loneliness should be cautiously interpreted and represents a conservative estimate of the underlying relationship. Although this binary approach facilitated comparison across relational domains, future studies should employ multi‐item scales that evaluate the intensity, motivation, and emotional tone of concealment in specific relationships. Such refinements would offer a more nuanced understanding of the role of concealment within everyday interactions, as well as ways of empirically capturing culturally specific meanings of silence.

Second, our sample was drawn from Tokyo Rainbow Pride 2015 and from a lesbian community space in a large metropolitan area, and thus primarily comprised urban‐living, community‐connected SGM women. Women who were more geographically isolated, disconnected from LGBTQ communities, or reluctant to attend public events were likely underrepresented. This sampling bias could underestimate the emotional burden of concealment among more isolated or rural SGM women and limits the generalizability of our findings beyond urban, community‐engaged populations. Recruitment in future studies should deliberately include participants from rural or less networked contexts to test the generalizability of these relational patterns.

Third, the data were collected between 2015 and 2016, which may limit the extent to which the findings reflect current social realities. Public discourse and institutional recognition of sexual and gender diversity in Japan have since gradually evolved, although progress remains uneven.^30^, ^31^ Consequently, the observed associations should be interpreted as context‐specific rather than generalizable to the present. Furthermore, although the data offer a snapshot of relational dynamics during the study period, their capacity to test current theoretical assumptions is inherently limited. Future studies employing more recent and longitudinal data are required to evaluate the persistence of similar relational patterns under changing social conditions.

Finally, the cross‐sectional study design limits causal interpretation. The associations identified here should be understood as exploratory and relational rather than deterministic.

Despite these limitations, the present study provides a robust foundation for subsequent work exploring the joint influence of relational contexts and cultural norms on the emotional meaning of concealment. Continued investigation across diverse samples, time periods, and methodological approaches will help clarify the dual function of silence as protection and as a source of psychological strain.

## CONCLUSION

This study suggests that concealment is not a uniform act of avoidance but a culturally embedded relational strategy, whose emotional costs vary by relational context. Concealment from friends appears more psychologically costly because it conflicts with norms of openness and mutual disclosure, whereas familial silence may function as an adaptive strategy for maintaining relational harmony. These findings highlight the importance of considering cultural scripts and relational norms when interpreting concealment behaviors. Future investigations should examine the impact of evolving sociocultural contexts on the meanings and consequences of concealment among SGM women in Japan.

## AUTHOR CONTRIBUTIONS

*Conceptualization*: Maya Hayakawa, Takeshi Asami, and Yuriko Tanabe. *Methodology*: Maya Hayakawa, Takeshi Asami, and Yuriko Tanabe. *Investigation and data curation*: Maya Hayakawa. *Formal analysis*: Maya Hayakawa and Yusuke Saigusa. *Resources*: Junichi Fujita, Akitoyo Hishimoto, and Yusuke Saigusa. *Visualization*: Maya Hayakawa. *Writing, original draft*: Maya Hayakawa. *Writing, review and editing*: Junichi Fujita, Akitoyo Hishimoto, Yusuke Saigusa, Takeshi Asami, and Yuriko Tanabe. *Supervision*: Junichi Fujita, Akitoyo Hishimoto, and Takeshi Asami. *Project administration*: Maya Hayakawa. Guarantor: Maya Hayakawa. All authors meet the criteria for authorship. Each author made substantial contributions to the work, participated in drafting or critically revising the manuscript, approved the final version, and agrees to be accountable for all aspects of the work.

## CONFLICT OF INTEREST STATEMENT

The authors declare no conflicts of interest.

## ETHICS APPROVAL STATEMENT

The study was approved by the Human Genome and Genetic Research Ethics Committee of Yokohama City University (Approval Nos. A151126016 and A190100005). Informed consent was obtained; completion of the anonymous, self‐administered paper questionnaire was regarded as provision of consent under the approved protocol.

## PATIENT CONSENT STATEMENT

Informed consent was obtained. Completion of the anonymous, self‐administered paper questionnaire was regarded as provision of consent under the approved protocol of the Human Genome and Genetic Research Ethics Committee of Yokohama City University.

## CLINICAL TRIAL REGISTRATION

Not applicable.

## Supporting information

Supporting Information.

## Data Availability

The datasets generated and analyzed during the current study are not publicly available because they contain potentially identifying information and may pose risks of social or religious harm. De‐identified data may be shared upon reasonable request to the corresponding author, Junichi Fujita, subject to approval by the Human Genome and Genetic Research Ethics Committee of Yokohama City University and a data use agreement. Study materials (e.g., the questionnaire and variable list) may be shared on request if permitted by the ethics approval.
