# Supplementary material for: Relationally specific sexual identity concealment and loneliness among sexual and gender minority women in Japan: A culturally situated analysis
Source: PCN Rep. 2026 Jan 8;5(1):e70280. doi: 10.1002/pcn5.70280 (PMC12783912; doi:10.1002/pcn5.70280)
Supplement: Supplementary file 1 — Supporting Information. [file PCN5-5-e70280-s001.docx]

|  |  | Concealment from parents | |  | Concealment from heterosexual friends | |  |
| --- | --- | --- | --- | --- | --- | --- | --- |
|  |  | Concealment 　(total N=95) M (SD) | Disclosure 　(total N=71) M (SD) | *P^*^* | Concealment 　(total N=34) M (SD) | Disclosure 　(total N=132) M (SD) | *P^*^* |
| Age | 18–19 y.o. (n = 13) | 39.6 (5.1) | 52.3 (15.5) | .29 | 41.2 (6.1) | 43.4 (11.5) | .67 |
|  | 20–29 y.o. (n = 93) | 39.2 (9.6) | 38.9 (11.3) | .88 | 45.0 (9.6) | 38.4 (10.4) | .12 |
|  | 30–39 y.o. (n = 33) | 41.8 (13.3) | 41.1 (13.0) | .87 | 51.3 (14.7) | 37.8 (10.2) | .03^*^ |
|  | Over 40 y.o. (n = 27) | 37.5 (10.2) | 43.5 (11.4) | .17 | 41.8 (9.1) | 37.1 (11.4) | .55 |
| Sexual orientation | Lesbian (n = 65) | 36.7 (9.4) | 38.9 (12.5) | .42 | 41.9 (14.1) | 37.1 (10.5) | .36 |
|  | Bisexual (n = 74) | 39.8 (10,5) | 44.3 (11.7) | .11 | 46.6 (10.8) | 39.8 (10.7) | .03^*^ |
|  | Queers^a^ (n = 17) | 45.0 (7.5) | 33.7 (9.2) | .03^*^ | 44.2 (8.4) | 37.4 (10.2) | .16 |
| Gender identity | Non-cisgender(n = 10) | 43.5 (9.2) | 41.8 (10.1) | .84 | - | 42.1 (9.5) |  |
| Other backgrounds | Single (n = 81) | 41.1 (10.0) | 43.3 (14.2) | .43 | 47.1 (11.6) | 40.6 (11.8) | .05 |
|  | Partnered (n = 85) | 37.9 (9.9) | 38.0 (8.8) | .95 | 42.1 (10.2) | 37.9 (9.0) | .05 |
|  | Substance use (n = 14) | 43.7 (13.3) | 45.6 (15.2) | .81 | 46.6 (13.9) | 43.6 (14.5) | .71 |
|  | Psychiatric service use (n = 50) | 44.7 (10.9) | 46.3 (10.4) | .58 | 49.4 (10.9) | 44.2 (10.3) | .13 |
|  | LGBTQ-based victimization(n=59) | 40.1(11.6) | 41.4(12.8) | .70 | 48.2(15.8) | 39.3(11.0) | .03^*^ |
|  | Perceived prejudice(n=154) | 40.4(10.7) | 40.5(12.1) | .96 | 46.6(11.6) | 38.8(10.6) | <.01^**^ |

Supplementary Table S1. Loneliness scores by concealment status (parents; heterosexual friends)

Note. Values represent mean loneliness scores (M) and standard deviations (SD) on the Japanese version of the Revised UCLA Loneliness Scale (R-UCLALS-J). Concealment and disclosure were coded dichotomously (yes/no) for each relational domain. “LGBTQ-based victimization” and “perceived prejudice” were coded as yes/no based on self-reported experiences. Independent samples t-tests were conducted to compare group means.
*p < .05, **p < .01.
